# Visual attention in bilingual instructional videos: effects of audiovisual congruency and subtitle language

**DOI:** 10.1186/s41235-026-00741-x

**Published:** 2026-06-16

**Authors:** Inka Romero-Ortells, Manuel Perea, Eva Gutierrez-Sigut, Jon Andoni Duñabeitia

**Affiliations:** 1 Centro de Investigación Nebrija en Cognición (CINC), Universidad Nebrija, Madrid, Spain; 2 Departamento de Metodología and ERI–Lectura, Universitat de València, Av. Blasco Ibáñez, 21, 46010 Valencia, Spain; 3 Department of Psychology, University of Essex, Colchester, UK

**Keywords:** Content comprehension, Gaze behavior, Language processing, Subtitles, Visual cues

## Abstract

How do subtitles shape where learners look, and what they learn, in bilingual instructional video? Subtitles are increasingly central to instructional video in multilingual education, where learners must integrate spoken input with written text. In the present paper, we recorded eye movements while university students watched two-speaker instructional videos to test the effects of audio language (original L2 vs L1 dubbed) and subtitle language (L1 vs L2). Experiment 1a (N = 40 Spanish L1, English L2) contrasted original English audio with Spanish-dubbed versions, and we found that dubbing shifted gaze from the speakers’ mouths to their eyes; comprehension was also higher for dubbed L1 than original L2. Experiment 1b tested the same Spanish-speaking participants with English-audio videos containing either English (L2) or Spanish (L1) subtitles, and included native English speakers viewing the English-audio/English-subtitle condition as a comparison group. In both groups, participants consistently devoted most of their gaze to subtitles rather than to the speakers’ eyes or mouths, independent of subtitle language; comprehension was equally high for L1 and L2 subtitles. Taken together, these findings indicate the key role of subtitles in guiding visual attention, with direct implications for the design of bilingual multimedia instruction.

## Introduction

Instructional videos have become a central component of modern education, supporting learning across various educational levels and disciplines (Brame, 2016; Giannakos, 2013; Laparra et al., 2023; Vieira et al., 2014). Unlike entertainment-oriented content, instructional videos are explicitly designed to promote knowledge transfer and actively engage viewers' cognitive processes. Their effectiveness depends not only on the quality of individual components (e.g., dialogue, images, and subtitles) but also on the learner’s ability to process and integrate information across these modalities (Clark & Paivio, 1991). The challenge of processing this information is particularly pronounced for second language (L2) learners, who must manage complex audiovisual input in a language they are still acquiring. This scenario is increasingly common as L2 education becomes central to curricula worldwide (Ernst & Bülthoff, 2004; Lalonde & Holt, 2016). The present study uses eye-tracking to investigate how the language of subtitles and audiovisual congruency influence learners' gaze allocation and comprehension in instructional videos.

The Cognitive Theory of Multimedia Learning (CTML; Mayer, 2014, Mayer, 2020, Mayer, 2024) proposes that learning is most effective when auditory and visual channels are coordinated to manage the limits of working memory (Ayres & Sweller, 2014; Paas & van Merriënboer, 2020; Sweller, 2005). For native speakers, the Redundancy Principle (Mayer, 2009; Mayer & Fiorella, 2014; Mayer & Pilegard, 2014) suggests that presenting identical information simultaneously (e.g., spoken words and on-screen text) can be redundant and distracting, thereby increasing cognitive load. In contrast, for L2 learners, this relationship is often reversed. The Subtitle Principle (Mayer et al., 2020), a core extension of the CTML, proposes that subtitles are not redundant but a crucial aid for processing spoken language. Unlike transitory audio, subtitles provide a stable visual representation of the spoken words, reducing the cognitive load associated with word recognition and speech perception in an unfamiliar language (Lee & Mayer, 2018). This distinction is particularly relevant in instructional contexts, where learners must integrate linguistic and conceptual information under task demands that differ from passive film viewing.

In any video, a learner's visual attention is naturally directed toward salient cues. In human interaction, this often includes the speaker's face, where the eyes convey emotional information (Birmingham et al., 2008; Itier et al., 2007) and the mouth provides articulatory cues that support speech perception (Buchan et al., 2007; Lalonde & Holt, 2016; Sumby & Pollack, 1954; Vatikiotis-Bateson et al., 1998; Võ et al., 2012; Wass & Smith, 2014). This multisensory integration, where audiovisual input creates a more robust signal, is a key element of CTML (Brünken et al., 2010; Mayer, 2014). However, attention to these cues is not fixed and varies depending on the task. Emotion-related tasks elicit more fixations on the speaker’s eyes (Chen et al., 2022; Emery, 2000), while speech processing increases fixations to the speaker’s mouth (Barenholtz et al., 2016; Lusk & Mitchel, 2016). Notably, Birulés et al. (2020) found a more balanced distribution of fixations between the eyes and the mouth when participants viewed videos in an L2. In their native language (L1) condition, viewers typically fixated on the eyes because of reduced need for articulatory cues during easier L1 speech processing (see also Grüter et al., 2023, for similar L1-L2 differences). For L2 learners, audiovisual incongruence (e.g., dubbed audio that does not match lip movements) can further alter these patterns, leading to a shift in gaze away from the mouth toward other cues such as the eyes (di Giovanni & Romero-Fresco, 2019; Romero-Fresco, 2020).

Beyond facial cues, subtitles serve as a powerful visual aid, directing attention and facilitating learning in multimedia environments. Subtitles are processed semi-automatically across the lifespan (d’Ydewalle et al., 1991; De Bruycker & d’Ydewalle, 2003) and, in L2 learners, subtitles increase comprehension, motivation, and vocabulary acquisition (Lertola, 2019; Talaván-Zanón, 2010), with L2 subtitles particularly aiding lexical development (Peters et al., 2016). In the context of L2 audio videos, both L1 subtitles (i.e., native-language translations) and L2 subtitles (i.e., same-language captions; Díaz-Cintas & Anderman, 2009) offer instructional benefits, including improved reading fluency, increased attentional engagement, and gains in pronunciation and literacy (Gernsbacher, 2015; Lei, 2023; To, 2024).

Research comparing subtitle languages has largely focused on behavioral outcomes such as comprehension (Lee & Mayer, 2018; Pannatier & Bétrancourt, 2024) or vocabulary learning (Wang & Pellicer-Sánchez, 2023). Fewer studies have examined how subtitle language shapes gaze allocation in instructional videos, despite evidence from film-based research showing that subtitle language modulates attention and incidental vocabulary learning (Bisson et al., 2014). Understanding gaze allocation is essential for linking audiovisual input to learning outcomes, particularly in L2 instructional contexts where learners must integrate linguistic and conceptual information in line with explicit learning goals.

To address this gap, we conducted an eye-tracking study composed of two closely related experiments (Experiment 1a and Experiment 1b) that tested how the alignment of auditory and visual input and the language of subtitles shape gaze allocation and comprehension in L2 instructional videos. We operationalized gaze behavior as proportional dwell time in different areas of interest (AOI: mouth, eyes, subtitles [Experiment 1b]). In combination, these experiments isolate complementary aspects of audiovisual learning in bilingual contexts.

Experiment 1a examined the role of audiovisual congruency. Prior research suggests that both audiovisual incongruence and relative ease of L1 processing can alter gaze patterns, shifting attention away from the mouth toward the eyes (Birulés et al., 2020; di Giovanni & Romero-Fresco, 2019; Romero-Fresco, 2020), whereas easier L1 speech processing may reduce reliance on articulatory cues and likewise increase attention to the eyes (see Birulés et al., 2020; Grüter et al., 2023). Because these accounts are not mutually exclusive, we compared videos with original English (L2) audio to those dubbed into the learner’s native language (Spanish, L1). We expected dubbed L1 videos to elicit relatively greater dwell time on the speakers’ eyes than on their mouths, whereas the original L2 videos were expected to produce a more balanced distribution of dwell time across the speaker’s mouth and eyes. This experiment established a baseline for testing whether audiovisual alignment influences gaze distribution in bilingual instructional videos, extending patterns observed in general viewing to educational contexts.

Experiment 1b, the key experiment, examined how subtitle language (Spanish as L1 vs English as L2) influences gaze behavior and comprehension during L2 audio–video viewing. Both formats are widely used in L2 learning (Black, 2022; Montero-Perez et al., 2014; Pérez-Serrano et al., 2021), yet few studies have directly compared their effects on visual attention, since most previous research has focused on measuring comprehension behaviorally (Lee & Mayer, 2018; Pannatier & Bétrancourt, 2024) or vocabulary learning separately (Wang & Pellicer-Sánchez, 2023). We tested both Spanish-speaking English L2 learners and native English viewers to isolate the effects of subtitle language using identical videos with synchronized audio, speakers’ facial cues, and subtitles. According to the Subtitle Principle within the Cognitive Theory of Multimedia Learning (Mayer et al., 2020), subtitles provide a stable visual representation of speech, directing attention to the text area. Given their salience and the cognitive demands of reading (d’Ydewalle et al., 1991), we predicted that participants, regardless of language background, would allocate most of their viewing time to the subtitle area in both conditions.

Furthermore, consistent with the Cognitive Load theory (Sweller, 2005) and the increased demands of less familiar linguistic input (Mayer, 2024), we hypothesized that the L2 subtitle condition would elicit greater proportional dwell time in the subtitle area, reflecting increased processing demands (Liao et al., 2020; Wang & Pellicer-Sánchez, 2023). Finally, we anticipated that comprehension accuracy would remain high in both conditions, consistent with prior research showing comparable learning outcomes for intermediate-to-advanced learners when subtitles are present (Pannatier & Bétrancourt, 2024). However, this may not generalize to lower-proficiency learners, as prior studies suggest that they may experience increased working memory load when processing L2 text (Hsieh, 2019; Suárez & Gesa, 2019). In such cases, L1 subtitles can provide more immediate semantic access (Pujadas & Muñoz, 2024) and often improve comprehension by reducing individual differences in working memory capacity and reading ability (Gass et al., 2019; Pujadas & Webb, 2025).

Taken together, the experiments test how audiovisual alignment and subtitle language (L1 vs L2) jointly shape visual attention and comprehension. The findings extend theoretical accounts of multimodal processing and provide evidence-based guidance for designing bilingual instructional materials.

## Experiment 1a: audiovisual congruency without subtitles

### Method

#### Participants

A total of 40 native Spanish speakers (23 women), aged 18–34 years (mean age = 21.8 years, *SD* = 3.7 years), from the Universitat de València (Spain), participated in the experiment. All participants had studied English as a foreign language since primary school (4 years in primary school and 6 years in secondary school). They reported their current levels of English following the last certificates they received—their current level ranged from intermediate (B1 [n = 12], B2 [n = 13]) to advanced (C1 [n = 13], C2 [n = 2]) following the Common European Framework of Reference for Languages: Learning, teaching, assessment (Council of Europe, 2001, 2020). Given the heterogeneity in English proficiency levels, we conducted exploratory analyses to examine whether proficiency could have modulated gaze patterns.

Participants reported normal (or corrected-to-normal) vision and no language-related disorders. All participants provided informed consent before the experiment began and received a small monetary compensation of €5 upon completion. The Ethics Committee of the Universitat de València approved the experiments. The participants were selected according to their native language, age range, and English proficiency criteria relevant to the goals of the study.

#### Materials

We created four videos with similar content. Each video focused on a single location: Belize, the Comoros Islands, Macao, or Tonga. We chose these places as they were relatively unknown to the participants. To ensure this, we searched (until April 2023) for some of the least-known places in the world, particularly from a Eurocentric perspective, and these four locations were frequently mentioned. In addition to their unfamiliarity, we chose these places for their geographical diversity, spanning Central America, the Indian Ocean, East Asia, and the South Pacific. This variety enables a broad comparison across cultures and regions. We selected five interesting facts about each place, the location, and an initial question that captured individuals’ curiosity. The vocabulary and structures used in the videos were adapted to the intermediate English level.

**Fig. 1:**
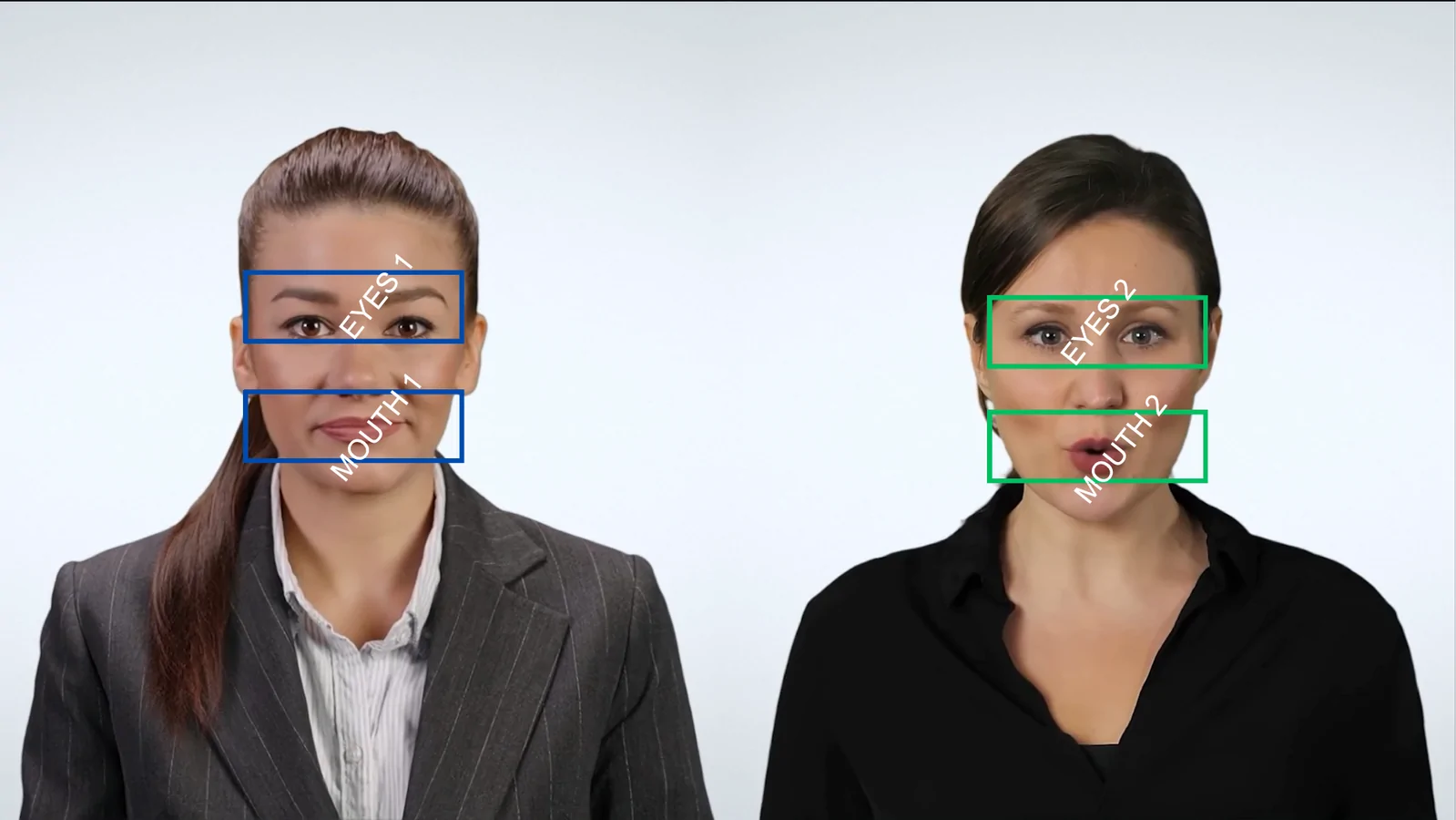
Screenshot of the distribution of the video and its AOIs in the different audio formats

Unlike previous studies that used a single speaker, our experiment featured two avatars to better simulate natural conversational dynamics. This design allowed us to examine how participants allocate visual attention across multiple speakers—a common real-world scenario in which both verbal and visual cues influence gaze shifts. Rather than limiting attention to a single speaker, the two-avatar setup captured how participants navigated speaker transitions, deciding whether to focus on the speaking avatar or the silent interlocutor. This approach enhances the ecological validity of our eye-tracking paradigm for studying conversational attention. Both avatars depicted professionally dressed women aged approximately 30–40 (see Fig. 1), and participants did not report noticing that the avatars were artificially generated.

We maintained the same distribution of characters in the videos to ensure that participants encountered similar stimuli and to reduce variability, with consistent English voices (spoken with a United States accent for better synchronization with mouth movements) for both characters. Likewise, we used consistent Spanish voices (with a Castilian accent, given that participants were recruited in Spain and used to this accent). All videos included historical, cultural, and language data and lasted 2 min and 2 s. We used the same script (see Appendix 1) for the English original (OV) and Spanish dubbed (DUB) versions. Stimuli can be checked through this OSF link (see videos with the OV_EN and OV_EN_DUB names):https://osf.io/m6792/?view_only=a5f72f8a71fb45a0b24376d15333bdc5

The videos were created using Synthesia® (2023) to select the avatars and voices and to generate initial audio–face synchronized versions from written scripts. In the version of Synthesia® used for this study, audio–face synchronization could only be inspected after video generation, and two avatars could not be displayed simultaneously. We therefore assembled the two-avatar videos in Adobe Premiere Pro® (2023). For the Spanish-dubbed condition, Spanish audio was generated in Synthesia® and then manually adjusted in Adobe Premiere Pro® by an experienced audiovisual translator. This adjustment followed established audiovisual-translation conventions, including the translation and adaptation of the content and the alignment of the dubbed speech with the visible articulatory and bodily movements of the on-screen speakers as closely as possible (Baños & Díaz-Cintas, 2018; Chaume, 2020).

Regarding the comprehension questions, participants answered 40 true/false statements, with ten questions per video and 1 point per correct answer (10 points per location) after watching the videos. These questions were designed to assess factual recall and comprehension of key information presented in each video. The questions focused on the opening statement, the geographical location, and the facts presented in the videos, ensuring comprehensive coverage of the videos' content. The true/false format was chosen for its clear assessment of specific knowledge points. Given that the videos could be viewed in Spanish or English, this instrument was presented in a bilingual format to facilitate reading and comprehension (i.e., *Belice es un paraíso de los buceadores*. / *Belize is a scuba diving paradise*). We assessed baseline familiarity in an additional group of individuals who had not seen the videos (N = 11); performance was close to chance (*M* = 22.55 out of 40, *SD* = 2.35). The comprehension questions can be found in the following OSF link: https://osf.io/m6792/?view_only=a5f72f8a71fb45a0b24376d15333bdc5

#### Procedure

Spanish-speaking participants completed Experiments 1a and 1b in a single session; they viewed four experimental videos corresponding to the two distinct parts of the study. The conditions are specified in Table 1.

**Table 1:** Video conditions viewed by each Spanish-speaking participant in Experiments 1a and 1b

Experiment part	Audio	Subtitles
1a	English (L2)	None
1a	Spanish (L1, dubbed)	None
1b	English (L2)	English (L2)
1b	English (L2)	Spanish (L1)

Experiment 1a involved the original English videos, whereas Experiment 1b involved the Spanish-dubbed videos. Each participant saw one video in each of the four conditions shown in Table 1, with the assignment of video topic to condition counterbalanced across participants

After participants signed the consent form, the experiment was conducted in a quiet laboratory. They were instructed to ask questions when anything about the procedure was unclear. Then, they moved to the eye-tracker-connected computer to proceed with the experiment, and the researcher was seated to the side to monitor them.

We employed a Windows 11 computer connected to a video-based eye-tracker, EyeLink© 1000 Plus (SR Research Ltd, Canada), with a sample rate of 1000 Hz. The videos were presented in a 1280 × 1024 resolution on an Asus VG248 144 Hz LCD 24″ monitor and a Windows computer running SR Research’s Experiment Builder program (version 2.4.1), with a high-definition headset (model Hercules HDP DJ60) at a comfortable listening level. EyeLink© Data Viewer (SR Research Ltd., Canada) was used to transform the data.

Participants were given simple written instructions displayed on the computer screen and asked to pay attention to the videos to answer comprehension questions. Because participants knew they would answer comprehension questions, viewing occurred under explicit learning goals rather than in a passive observation mode. They were seated 60 cm away from the monitor and used a chin and forehead rest to minimize movements. Data were collected from the participant’s right eye.

#### Eye-tracking acquisition and preprocessing

Gaze was recorded monocularly (right eye) at 1000 Hz using the EyeLink 1000 Plus. A 9-point calibration/validation was completed at the start, and drift correction was performed before each trial using a central fixation target. Saccades were detected with EyeLink default thresholds (velocity 30°/s, acceleration 8000°/s^2^). Blinks and host-flagged invalid samples were removed; short gaps (< 75 ms) were linearly interpolated. Fixations shorter than 60 ms or longer than 1200 ms were excluded. Trials with more than 25% track loss or calibration error greater than 0.5° were discarded a priori.

To quantify selective attention, we created areas of interest (AOIs) around the eyes (322 × 122 mm) and mouth (322 × 122 mm) (see an example in Fig. 1). Using Data Viewer, the data were transformed into area-of-interest reports that included dwell time within each AOI (in ms) and total valid trial dwell time for the trial (in ms). These measurements were chosen because dwell time, representing the cumulative duration of fixations within an AOI, is a robust indicator of sustained visual attention and cognitive processing directed toward specific visual features (e.g., the eye region for social cues, the mouth region for speech processing). The denominator was the total valid viewing time in the trial after preprocessing, including valid gaze samples that fell outside the predefined AOIs. Calculating the ratio of AOI dwell time to total valid viewing time allowed us to normalize individual differences in overall viewing patterns and to quantify the proportional allocation of attention to the eyes and mouth regions. This approach examines how visual attention is distributed across key facial features under various language conditions, offering insights into the attentional demands of processing speech in L1 versus L2.

Each participant began with a 30-s instructional video, shown only once. The practice video had the same format as the first video shown to participants. The trial videos depended on the condition-counter-balanced list to which the participant was assigned. After each video, the corresponding comprehension questions were presented on a Microsoft Surface Go 10 + , and participants were instructed to answer them as accurately as possible.

#### Data analysis preparation

We analyzed the cumulative time (dwell time, in ms) spent in each region of interest (AOI) for two avatars displayed on the screen under two conditions: English (L2) original audio and Spanish (L1) dubbed audio. First, we combined the repeated AOI dwell times and converted them from milliseconds to seconds. Next, we calculated the overall dwell time for the AOIs (eyes and mouth separately). We then divided each AOI dwell time by the total valid dwell time in the trial to determine the ratios for the eyes and mouth:

- Eyes ratio = eyes dwell time / total valid viewing time
- Mouth ratio = mouth dwell time / total valid viewing time

This methodology follows the approach employed in previous studies (Barenholtz et al., 2016; Birulés et al., 2020; Lusk & Mitchel, 2016), which have similarly utilized these metrics to examine attention allocation in dynamic social stimuli. We conducted these computations for both L2 audio and L1 audio. JASP (version 0.17.1.0) (Wagenmakers et al., 2018) was used to run statistical analyses.

### Results and discussion

**Fig. 2:**
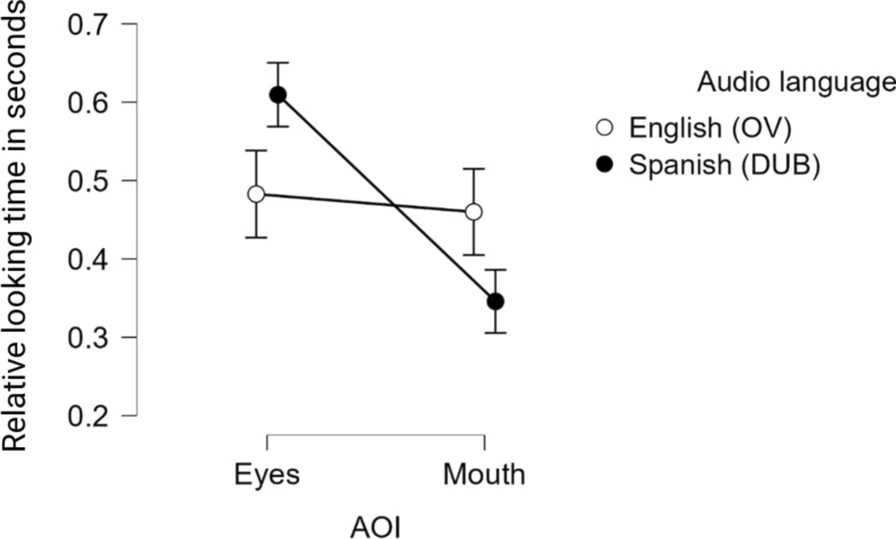
Proportional dwell time for the eyes and mouth in the original versus dubbed version. The bars represent the standard error of the mean

We tested whether the video's audio language (original English [L2] versus dubbed Spanish [L1]) influenced the amount of time participants allocated to the speaker’s eyes and mouth. To do this, we conducted a 2 (AOIs: eyes versus mouth) × 2 (Audio language: English [OV] versus Spanish [DUB]) Analysis of Variance (ANOVA) on the mean proportional AOI dwell times, with both factors treated as within-subjects variables. Figure 2 shows each AOI’s proportional dwell time across both languages. The analysis can be found in the following OSF link (see “Spanish group” analysis): https://osf.io/m6792/?view_only=a5f72f8a71fb45a0b24376d15333bdc5

While the main effects of AOI and audio language were not statistically significant ([*F*(1,39) = 2.47, *p* = .124] and [*F*(1,39) = 3.32, *p* = .076], respectively), we found a significant interaction between the two factors [*F*(1,39) = 16.99, *p* < .001]. Simple effects analyses revealed that, under the Spanish-dubbed (L1) audio condition, participants spent a longer proportion of time on the speakers’ eyes than on their mouths (see Table 2), *F*(1,39) = 14.17, *p* < .001. In contrast, under the English-original (L2) audio condition, these proportions were nearly identical, *F*(1,39) = 0.05, *p* = .813. Another way to interpret this interaction is to examine the effect of audio language on each AOI. Participants spent a longer proportion of time on the speakers’ eyes during the Spanish-dubbed (L1) version than during the English-original (L2) version, *F*(1,39) = 17.21, *p* < .001. Conversely, participants spent more time looking at the speakers' mouths during the English-original (L2) audio condition compared to the Spanish-dubbed (L1) version, *F*(1,39) = 16.16, *p* < .001.

**Table 2:** Proportional dwell times on the eyes and the mouth AOIs by audio language

Audio language	AOI	N	Mean	SE
English (OV)	Eyes	40	0.483	0.049
	Mouth	40	0.460	0.047
Spanish (DUB)	Eyes	40	0.609	0.036
	Mouth	40	0.346	0.035

Legend: AOI = Area of Interest; OV = Original Version; DUB = Dubbed. This table shows the mean proportional dwell times and standard errors (SE) for participants' gazes on the eyes and mouth in both the original English and dubbed Spanish conditions. Data from 40 participants were analyzed for each condition. The proportional dwell time represents the fraction of total valid viewing time spent on a specific area. Note that proportions do not sum to 1.00 because gaze spent outside these AOIs contributed to the denominator (total valid sample time) but is not shown in the table

Although not the primary focus of the experiment, we also analyzed participants’ performance on the 10-question comprehension survey. Overall, the scores were reasonably good and showed higher comprehension of the Spanish-dubbed (L1) version (*M* = 8.93) than of the English-original (L2) version (*M* = 8.15), *t*(39) = -2.716, *p* = .010.

We then tested whether English proficiency modulated gaze allocation. We conducted an additional mixed ANOVA including participants’ most recently certified English level (B1–C2) as a between-subjects factor. This analysis revealed no statistically significant effects involving certified English level: the main effect of certified level was not significant, *F*(3,36) = 0.300, *p* = .825, nor were the Audio Language × certified level interaction, *F*(3,36) = 0.079, *p* = .971, the AOI × certified level interaction, *F*(3,36) = 0.593, *p* = .624, or the three-way interaction between Audio Language, AOI, and certified level, *F*(3,36) = 0.956, *p* = .424. Thus, the gaze patterns observed in Experiment 1a do not appear to have been modulated by English proficiency, as indexed by participants’ certified proficiency level.

Experiment 1a showed that participants who watched instructional videos dubbed into Spanish (L1) spent more time looking at the speaker’s eyes than at the speaker's mouth. In contrast, during the original English (L2) version, viewing time was more evenly distributed between the eyes and mouth. Comprehension scores were also higher for the dubbed Spanish version compared to the original English version.

These results are consistent with two non-mutually exclusive interpretations. First, audiovisual misalignment in dubbed speech may reduce the informativeness of the mouth, thereby shifting attention toward other socially salient features such as the eyes (di Giovanni & Romero-Fresco, 2019; Romero-Fresco, 2020). Second, L1 speech processing is less demanding, reducing the need to consult articulatory cues and naturally increasing attention to the eyes (Birulés et al., 2020; Grüter et al., 2023). Our findings extend these accounts to instructional videos, a context in which the primary goal is knowledge transfer.

## Experiment 1b: Subtitles in English-audio videos

Building on Experiment 1a, which addressed audiovisual congruency, Experiment 1b focused on subtitles as a complementary source of visual–linguistic input in instructional videos. Specifically, we asked how Spanish (L1) and English (L2) subtitles affected visual attention to the speakers’ faces and the subtitle area, and how they influenced comprehension.

Based on the Cognitive Theory of Multimedia Learning (Mayer, 2024), we hypothesized that both subtitle types would support comprehension to a similar extent, particularly given our participants’ intermediate-to-advanced L2 proficiency (Mayer et al., 2020). However, we predicted that participants would allocate relatively more dwell time to subtitles in the L2 condition (English) due to the greater processing demands associated with less familiar linguistic input (Liao et al., 2020; Muñoz-Lahoz, 2017; Sweller, 2014). To provide a baseline for gaze allocation patterns, we also included a group of native English speakers, allowing us to determine whether subtitle effects were specific to L2 processing or reflected more general attentional mechanisms.

### Method

#### Participants

The Spanish-speaking participants in Experiment 1b were the same participants tested in Experiment 1a and completed the subtitle conditions within the same session. For comparison, we recruited 24 native English speakers (16 women), aged 18–24 (mean = 20.5 years, *SD* = 1.47 years), from the University of Essex (United Kingdom). Participants reported normal (or corrected-to-normal) vision and no language-related disorders. All participants provided informed consent before the experiment, and native English speakers received optional curricular credits as compensation for their participation. The Ethics Committee of the Universitat de València and the University of Essex approved this experiment.

#### Materials and procedure

English and Spanish subtitles were added in Adobe Premiere Pro® (2023), as specified in Table 1. Subtitles were placed at the bottom center of the screen and synchronized with the spoken text following audiovisual translation standards (Díaz-Cintas, 2019). The subtitling was done according to established translation rules: appearing at the bottom-center of the screen, with a maximum of two lines and 42 characters per line, a sans serif font, for 4 to 6 s synchronized with the audio (see Díaz-Cintas, 2019; Díaz-Cintas & Anderman, 2009; Díaz-Cintas & Orero, 2010, for more specific characteristics of the subtitles). An experienced audiovisual translator ensured that the video versions met the standards of audiovisual translation. Stimuli can be checked through this OSF link (see videos with the OV_EN_EN SUBS and OV_EN_EN_ES SUBS names): https://osf.io/m6792/?view_only=a5f72f8a71fb45a0b24376d15333bdc5.

**Fig. 3:**
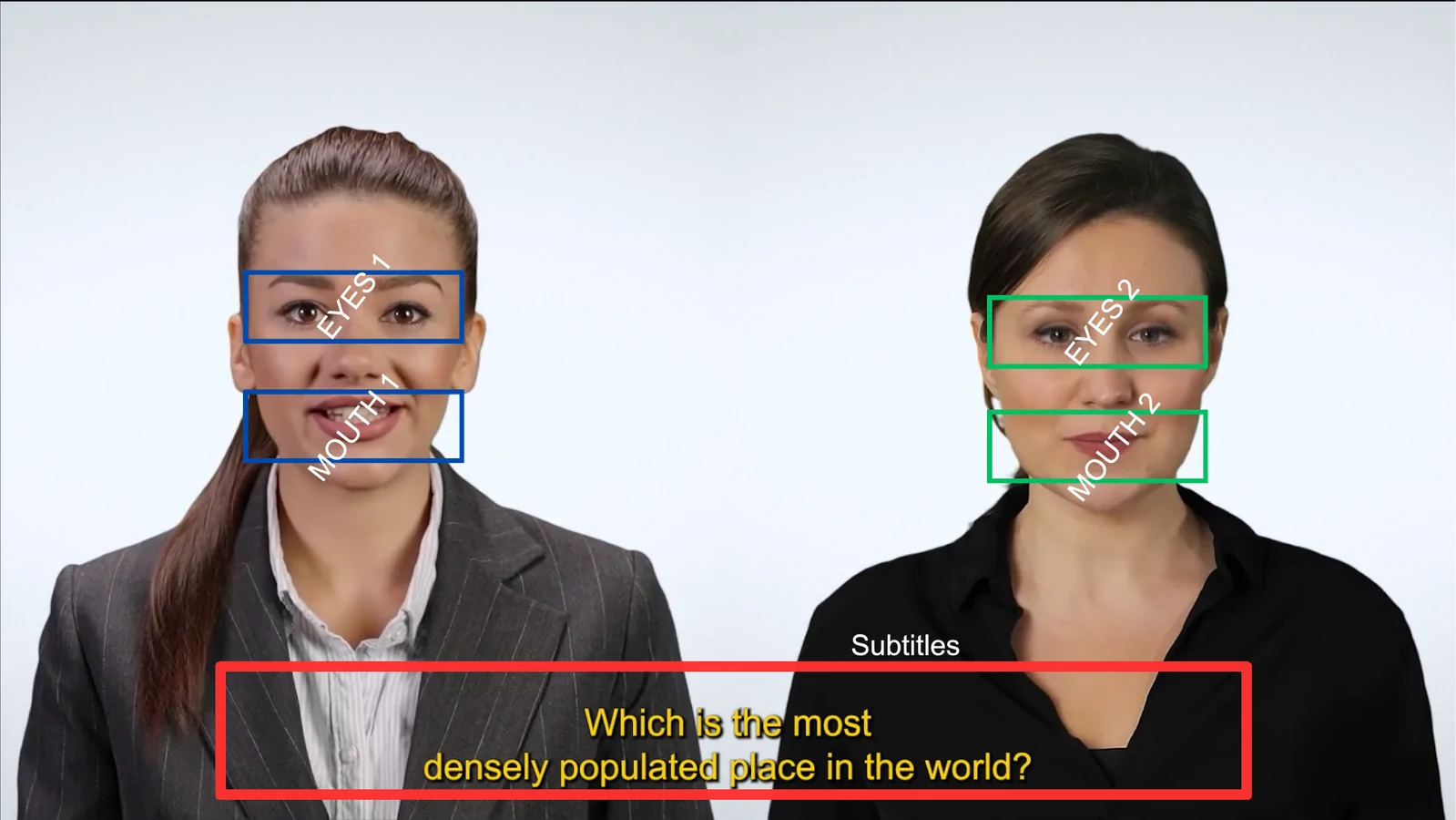
Screenshot showing the eye, mouth, and subtitle AOIs in the subtitle video conditions

To quantify selective attention, we created areas of interest (AOI) around the eyes (322 × 122 mm), mouth (322 × 122 mm), and subtitle area (1284 × 140 mm) (see Fig. 3). Through Data Viewer, the data were transformed into area-of-interest reports with the following variables: dwell time (in ms) and total trial dwell time.

#### Data analysis preparation

Following the same logic as Experiment 1a, we analyzed the cumulative dwell time in each AOI for the two conditions: English audio with English subtitles and English audio with Spanish subtitles. First, we combined the AOI dwell times and converted them from milliseconds to seconds. Next, we calculated the overall dwell time for the AOIs (eyes, mouth, and subtitles). We then divided each AOI dwell time by the total valid viewing time in the trial to determine the ratios for the eyes, mouth, and subtitles:

- Eyes ratio = eyes dwell time / total valid viewing time
- Mouth ratio = mouth dwell time / total valid viewing time
- Subtitles ratio = subtitles dwell time / total valid viewing time

### Results and discussion

This experiment tested how the presence and language of subtitles influence visual attention and comprehension during L2 instructional video viewing. We conducted two sets of analyses. First, we examined Spanish-speaking L2 learners who watched English videos with either Spanish (L1) or English (L2) subtitles, focusing on gaze allocation to the eyes, mouth, and subtitle areas of interest (AOIs). Second, we analyzed the gaze patterns of native English speakers who viewed the same English videos with English subtitles, providing a baseline for general subtitle processing. The analysis can be found in the following OSF link (see “Spanish group”, “English group”, “Groups” analysis): https://osf.io/m6792/?view_only=a5f72f8a71fb45a0b24376d15333bdc5

For the Spanish group, we examined the proportional dwell time in the AOIs (eyes/mouth/subtitles) relative to the total valid viewing time for L2 subtitles (original video in English with English subtitles) and L1 subtitles (original video in English with Spanish subtitles). We conducted a 3 (AOIs: eyes, mouth, subtitles) × 2 (Subtitle language: English-L2 versus Spanish-L1) Analysis of Variance (ANOVA) on the proportional AOI dwell time, treating both factors as within-subjects variables.

**Fig. 4:**
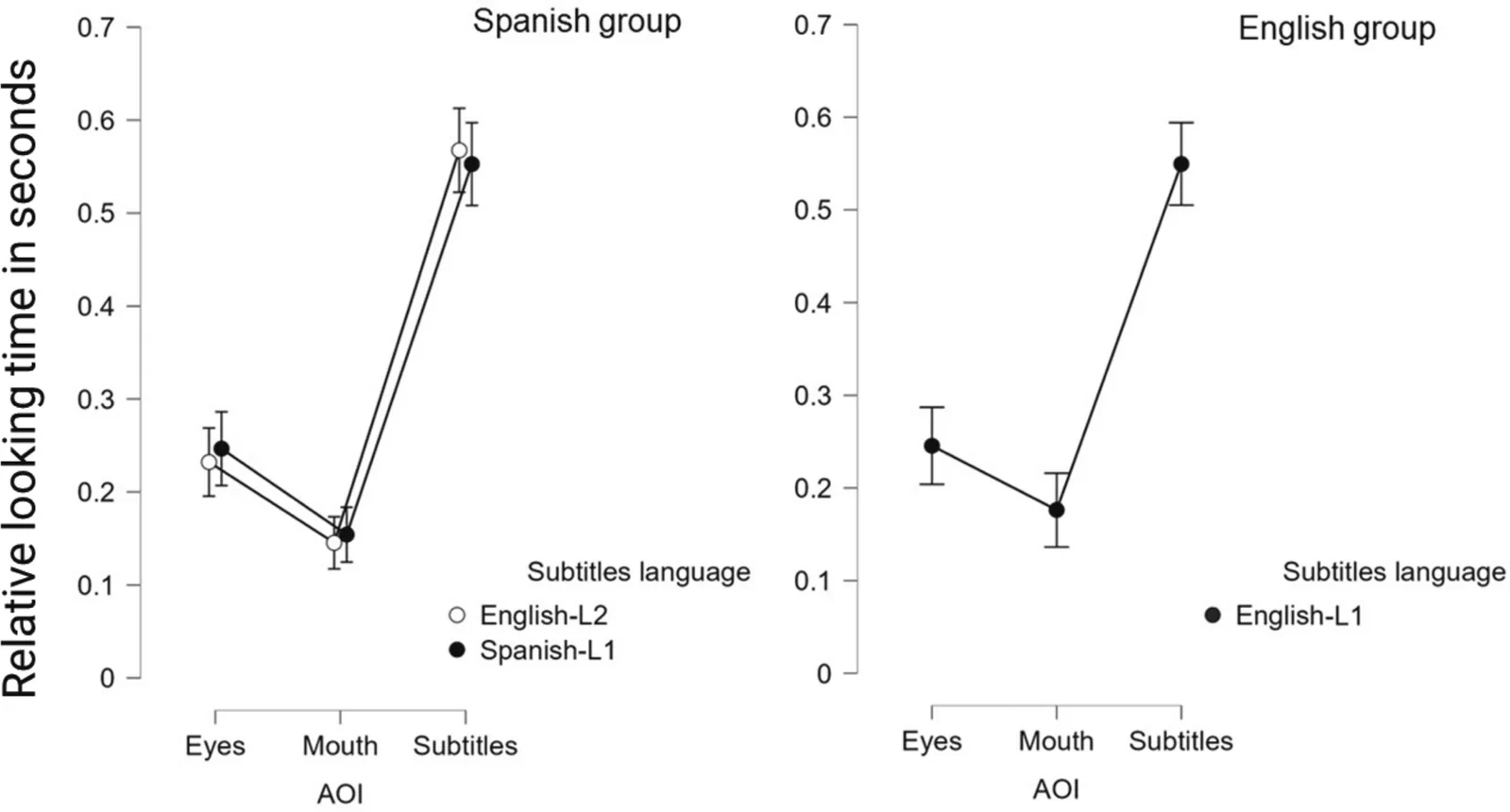
Proportional dwell time for eyes, mouth, and subtitles in the Spanish group (left panel) and the English group (right panel). The bars represent the standard errors of the means

The ANOVA showed significant differences in proportional dwell time across AOIs (*F*(2, 78) = 27.505, *p* < .001). This effect reflected, after adjusting the p-values with the Holm-Bonferroni procedure to correct for multiple comparisons, longer dwell time on the subtitles (see Table 3) than on the speakers’ eyes (*t*(39) = -5.513, *p* < .001), or mouth (*t*(39) = -7.053, *p* < .001)—the proportional dwell time did not differ between the speaker’s eyes and mouth (*t*(39) = 1.541, *p* = .127). Interestingly, the gaze pattern was remarkably similar across the L2 and L1 subtitle conditions, *F*(1,39) = 1.148, *p* = .290, with comparable dwell times in both conditions. Furthermore, there was no interaction between AOI and subtitle language (i.e., the patterns of dwell times for subtitles and the speakers’ eyes and mouths were similar for L1 and L2 subtitles; see Fig. 4), *F*(2,78) = .660, *p* = .520.

**Table 3:** Proportional dwell times on the eyes, mouth, and subtitles by subtitle language

Subtitle language	AOI	N	Mean	SE
English-L2	Eyes	40	0.232	0.033
	Mouth	40	0.145	0.025
	Subtitles	40	0.568	0.042
Spanish-L1	Eyes	40	0.247	0.036
	Mouth	40	0.154	0.027
	Subtitles	40	0.553	0.041

Legend: AOI = Area of Interest; L1 = Native Language; L2 = Second Language. This table presents the mean proportional dwell times and standard errors (SE) for participants' gazes on the eyes, mouth, and subtitles in original English (L2) videos, comparing conditions with either English (L2) or Spanish (L1) subtitles. Proportional dwell time refers to the fraction of total valid sample time spent on a specific area of interest. Data are from 40 participants in each subtitle condition. Note that proportions do not sum to 1.00 because gaze samples falling outside these AOIs contributed to the denominator but are not shown in the table

We also examined Spanish participants’ performance on the comprehension questions with L1 or L2 subtitles. A paired-samples t-test showed that comprehension scores were high and very similar between the two conditions (L1 subtitles: *M* = 8.90; L2 subtitles: *M* = 8.75), and the difference was not statistically significant, *t*(39) = –0.73, *p* = .467. To further evaluate this null effect, we conducted a Bayesian paired-samples t-test. This analysis quantified evidence for the null hypothesis (no difference in comprehension between L1 and L2 subtitles) relative to the alternative hypothesis. Using a non-informative Cauchy prior with a mean of 0 and a scale of 0.707, the analysis showed that the data were 4.56 times more consistent with the null hypothesis than with the alternative (*BF*_₀₁_ = 4.56, *error%* = .047). Thus, both the eye-tracking and comprehension data converge in suggesting that L1 and L2 subtitles yielded highly similar outcomes in this experiment.

As an additional exploratory analysis, we compared the comprehension scores from this experiment to those from Experiment 1a, specifically the English-original (L2) condition without subtitles. Results showed lower comprehension scores without subtitles (*M* = 8.15) than with either L1 subtitles (M = 8.90) (*t*(39) = -3.167, *p* = .003), or L2 subtitles (M = 8.75) (*t*(39) = -2.243, *p* = .031). Therefore, subtitles, whether in L1 or L2, improve L2 learners' comprehension of instructional videos.

As in Experiment 1a, to examine whether English proficiency modulated gaze allocation, we conducted an exploratory mixed ANOVA including the participants’ most recently certified English level (B1–C2) as a between-subjects factor. These exploratory analyses revealed no significant effects involving subtitle language and certified level: neither the Subtitle Language × certified level interaction nor the three-way interaction with AOI reached significance (all *ps* > .50). Although the AOI × certified level interaction was statistically significant, *F*(6,72) = 2.528, *p* = .028, this effect did not interact with subtitle language and appeared to reflect only minor variations in how different certified-level groups distributed attention across the three AOIs. Consistent with this interpretation, the main effect of certified level was not significant, *F*(3,36) = 0.579, *p* = .633. Post hoc comparisons (Holm-corrected) showed that, although B1 learners tended to allocate slightly more attention to subtitles than higher-level groups, none of these differences survived correction, and they did not interact with subtitle language (all *p*_Holm_ > .06). Thus, the observed gaze pattern appears to have been quite similar across participants’ reported certified English levels.

For comparative purposes, we also examined the gaze patterns of native English speakers watching videos with English audio and subtitles. For this purpose, we conducted an ANOVA with AOI (eyes, mouth, subtitles) as a within-subjects factor, revealing a pattern strikingly similar to that observed in the Spanish L2 learners. Figure 4 shows the proportional dwell time for each AOI in the two Spanish-group subtitle conditions (English-L2 and Spanish-L1) and in the native-English comparison condition (English-L1).

Similarly to the Spanish participants, the dwell time differed across the three AOIs [*F*(2, 46) = 22.369, *p* < .001], reflecting longer dwell time on the subtitles area (*M* = .550) relative to the speakers’ eyes (*M* = .245; *t(*23) = -5.120, *p* < .001) and mouth (*M* = .176; *t*(23) = -7.975, *p* < .001). Again, there were no significant differences between proportional dwell time for the speaker’s eyes relative to their mouth (*t*(23) = 1.167, *p* = .249).

Finally, we conducted a joint analysis of the proportional dwell times for the English subtitles across both the Spanish L2 and native English groups, adding Group as a between-subject factor. As shown in Fig. 4, the overall pattern was similar across groups (effect of group: *F*(1, 62) = 2.96, *p* = .090; interaction group × AOI: *F* < 1). Unsurprisingly, the analysis revealed that the dwell time differed across the three AOIs [*F*(2, 124) = 44.970, *p* < .001], reflecting longer proportional dwell time on the subtitle area relative to the speakers’ eyes (*t*(62) = -7.193, *p* < .001) and mouth (*t*(62) = -8.949, *p* < .001). Again, we found no significant differences between proportional dwell time for the speakers’ eyes relative to their mouth (*t*(62) = 1.756, *p* = .082).

In sum, Experiment 1b showed that native Spanish speakers watching English instructional videos with subtitles devoted most of their visual attention to the subtitle area rather than to the speaker’s facial features (eyes or mouth). This pattern was consistent across both subtitle languages (L1 Spanish and L2 English). The same pattern was observed in the native English group, whose participants showed the same gaze preferences when viewing the videos with English subtitles. This pattern aligns with findings from film-based research showing that subtitles strongly attract visual attention regardless of language (Bisson et al., 2014).

Comprehension scores for Spanish L2 learners were high and did not differ significantly between L1 and L2 subtitles, indicating that subtitle language did not affect understanding. Both subtitles, however, supported better comprehension than viewing without subtitles. These findings suggest that subtitles reliably guide learners’ attention and facilitate comprehension in multimedia learning, with little evidence that this pattern was modulated by subtitle language or certified L2 proficiency level. Nonetheless, given that our sample consisted of intermediate-to-advanced L2 learners, these similarities may not generalize to lower proficiency levels.

## General discussion

The present experiments investigated how audiovisual congruency and subtitle language affect gaze behavior and comprehension in instructional videos. The results indicate that learners allocate attention to the most reliable sources of information, thereby refining predictions from the Cognitive Theory of Multimedia Learning (CTML; Mayer, 2014,Mayer, 2020,Mayer, 2024), with implications for L2 instructional design.

Experiment 1a showed that the usefulness of a visual channel depends on its reliability. With original English (L2) audio synchronized to lip movements, participants divided their gaze between the mouth and the eyes. This distribution is consistent with multisensory integration in CTML (Mayer, 2014), where auditory and visual speech cues are combined to support processing. When the audio was dubbed into the native language (L1), gaze shifted from the mouth to the eyes. This pattern is consistent with two non-mutually exclusive interpretations: audiovisual mismatch may have reduced the informativeness of the mouth (di Giovanni & Romero-Fresco, 2019; Romero-Fresco, 2020), and the lower processing demands of L1 speech may have reduced learners’ reliance on articulatory cues (Birulés et al., 2020; Grüter et al., 2023). The shift may reflect adaptation to both signal reliability and processing demands in an instructional scenario. In addition, the higher comprehension scores in the dubbed L1 condition suggest a trade-off: original L2 audio may provide richer exposure to the target language, whereas dubbed L1 audio supports more accurate content comprehension. Importantly, instructional videos differ from entertainment media in their task demands, as learners view them with the explicit expectation of understanding and retaining information. This goal-directed context likely encouraged participants to prioritize reliable linguistic cues and may have amplified the shift away from the mouth in the dubbed condition.

Experiment 1b examined how subtitle language—L1 (Spanish) or L2 (English)—affects gaze and comprehension. Participants, both L2 learners and native English speakers, spent most of their viewing time on the subtitles rather than on the speaker’s face. This preference supports the Subtitle Principle (Mayer et al., 2020). It shows that subtitles serve as the primary source of linguistic information, regardless of viewers' proficiency in the language or the audio's congruency. Gaze analyses further revealed that subtitles, whether in L1 or L2, consistently reduced dwell time on the speaker’s mouth. Contrary to our prediction, the greater processing demands of L2 subtitles did not result in longer dwell times than those of L1 subtitles. This finding suggests that for learners with intermediate-to-advanced proficiency, both subtitle types are sufficiently accessible to act as stable visual anchors. The similarity of gaze patterns and comprehension across groups suggests that subtitle use is not entirely automatic. Instead, it appears to reflect a semi-automatic process (d’Ydewalle et al., 1991; de Bruycker & d’Ydewalle, 2003) that is efficient and readily engaged, but still adaptable to changes in task goals and information reliability (Pannatier & Bétrancourt, 2024; Tarchi & Mason, 2022). Learners appear to prioritize subtitles as the most reliable channel for linguistic input, facilitating dual-channel processing (Clark & Paivio, 1991) and supporting comprehension of complex material. This interpretation aligns with findings from film-based research showing that subtitles reliably attract visual attention and can shape linguistic processing even when viewers are not explicitly focused on language learning (Bisson et al., 2014).

Our findings provide practical guidance for designing instructional videos. Subtitles should be considered a central visual support that directs learners’ attention to essential content. For educators and content creators, this means ensuring subtitle clarity, prominence, and synchronization across languages, so that key information remains accessible. The results also show that for instructional materials with non-native audio, both L1 and L2 subtitles improve comprehension compared to audio alone. By providing a stable, easily processed source of information, subtitles help manage cognitive load, allowing learners to focus on meaning rather than decoding speech. However, while subtitles support content learning, they may not always align with goals centered on improving L2 speech perception. In instructional contexts aimed at strengthening listening skills, educators may therefore wish to use subtitles strategically (e.g., by combining captioned viewing with activities that redirect attention to the acoustic signal).

Content creators should also recognize that subtitles can draw attention away from a speaker’s face and reduce reliance on articulatory cues. While this trade-off is advantageous for most content-focused videos, it may require complementary strategies in pronunciation-oriented instruction, where facial movements are important. Because subtitles often become the primary visual focus, their legibility, timing, and accuracy are critical. Treating subtitles as integral design elements, rather than peripheral aids, can improve the effectiveness and accessibility of instructional videos.

While our study provides valuable insights, it also opens several avenues for future research. First, our L2 learners were Spanish university students with intermediate to advanced English proficiency. Learners with lower proficiency may show different gaze patterns and comprehension outcomes, perhaps relying more on L1 subtitles under high cognitive load or spending more time on L2 subtitles when working to strengthen reading skills. Second, our videos excluded gestures, diagrams, and other visual cues. Although this simplification reflects many higher-education materials, future work should examine whether the dominance of subtitles persists when additional nonverbal information is available. Finally, although both L1 and L2 subtitles improved comprehension, we did not assess their distinct effects on long-term language learning. L2 subtitles may provide added benefits by reinforcing mappings between spoken and written forms, a key mechanism for vocabulary growth and fluency. Future studies should investigate how sustained exposure to subtitles influences lexical development and broader language outcomes across diverse learner populations. This question is particularly relevant, as subtitles can facilitate incidental vocabulary learning during naturalistic viewing (Bisson et al., 2014; Pérez-Serrano et al., 2021), suggesting linguistic improvement beyond immediate comprehension.

In summary, our findings indicate that subtitles play a crucial role in directing visual attention and facilitating comprehension during bilingual multimedia learning. Rather than acting as peripheral aids, subtitles function as stable visual anchors that shape how learners engage with multimodal input. Our findings suggest that viewers allocate attention according to the reliability and usefulness of available information: when audiovisual speech is aligned, the mouth remains an informative source; when speech is dubbed, the mouth becomes less informative and gaze shifts toward the eyes; when subtitles are present, written text becomes the dominant anchor. This pattern refines predictions from the Cognitive Theory of Multimedia Learning by showing that redundancy depends on the learner, the task, and the reliability of the available channels. The design implication is clear: subtitles should be treated as integral components of instructional video design rather than optional add-ons.

## Data Availability

The materials are presented in Appendix 1. The data, scripts, and output files are available at the following OSF link: https://osf.io/m6792/?view_only=a5f72f8a71fb45a0b24376d15333bdc5

## Appendix 1

### Belize script

Where is the scuba diving paradise?

Belize is also known to be a scuba diving paradise and has a perfect harmony with nature, with 120 protected areas.

Would you like to know more?

Keep watching!

Belize is a Caribbean and Central American country on the north-eastern coast of Central America.

It is bordered by Mexico to the north, the Caribbean Sea to the east, and Guatemala to the west and south.

It also shares a water boundary with Honduras to the southeast.

We could talk for an hour about it but let us keep it short with five interesting facts.

Did you know that Belize is the only country in the world with a nature sanctuary dedicated to preserving great cats?

The sanctuary, near the Maya Mountains, is home to jaguars and pumas, and there is also a large population of mammals and birds to support the food chain.

Hundreds of years before the arrival of European colonists, the Maya Empire stretched for over a thousand miles in what is now Central America.

Today’s Belize was at the center of all the most important Maya trade routes.

What language do they speak?

A British colony for over 200 years, Belize is today the only nation in Central America where English is the official language.

Do they have a special place to dive?

In 1971, famed marine biologist Jacques Cousteau discovered what had long been the sole province of local fishermen, the Great Blue Hole, a unique diving site in the middle of an offshore coral atoll.

The Great Blue Hole is regularly voted as one of the Top 10 best diving sites in the world.

Moreover, as part of the second-largest barrier reef system in the world, only Australia’s Great Barrier Reef is bigger; the Belize Barrier Reef is an incredibly diverse marine ecosystem stretching for more than 180 miles off the coast of Belize.

Would you like to know more?

Go to the Belize Tourism Board and discover it!

### Comoros script

Can we see a lunar landscape without taking a rocket?

The answer is yes.

The Comoros are known as the moon islands, as the medieval Arabic geographers established the similarity when they arrived and saw the petrified lava above the white sand beaches.

Would you like to know more?

Keep watching!

The Comoros are an Indian Ocean archipelago situated near the southeastern coast of Africa, between Mozambique and Madagascar.

The Union of Comoros is formed by three principal islands, Anjouan, Mohéli, and Grande Comore, but the Comoros reclaims sovereignty over a fourth island, Mayotte, under French administration.

We could talk for an hour about it, but let us keep it short with five interesting facts.

Did you know that the Comorians are an Arabic-African ethnic mix group?

Most of the population is of an Arab-Islamic culture. That is why many carpets have a compass in one of the ends to know where Mecca is and to pray.

The first confirmed human inhabitants of the Comoros are believed to be of Malayo-Polynesian descent, arriving by the 5th or sixth century C.E. The first Europeans known to have visited the Comoros are thought to have been the Portuguese during the late sixteenth century.

What language do they speak?

The most common languages are Arabic and Comorian, a language closely related to Swahili; nevertheless, most of the population understands and speaks French.

Why are the Comoros well-known for?

It is known as the essence country, because some of the most expensive perfumes in the world are produced here.

In fact, Comoros is the world’s largest producer of the ylang-ylang flower, supplying around 80% of the world’s ylang-ylang flowers.

And did you know that Moroni, the country's capital, has only a traffic light?

You can imagine how crazy it would be driving there.

Would you like to know more?

Go to the National Tourism Office of the Comoros and discover it!

### Macao script

Which is the most densely populated place in the world?

While other places might seem more overpopulated, the key to Macau is its tiny size.

A population of 648,500 people squeezes into just 30.5 square kilometers, making it the most densely populated territory on earth.

Would you like to know more?

Keep watching!

Macau is a city and a special administrative region in China. It is in the south of Guangdong Province, on the tip of the peninsula formed by the Zhujiang (Pearl River) estuary on the east and the Xijiang (West River) on the west.

We could talk for an hour about it but let us keep it short with five interesting facts.

Did you know that Macau was the last European colony in China?

It was leased to Portugal in 1557 as a trading post and officially became a Portuguese territory in 1887.

But it was eventually handed back to Chinese sovereignty in 1999.

What language do they speak?

Patuá is a creole language, meaning that it is a blend of Portuguese and Cantonese, and developed in Macau to become the language of Macau’s indigenous Eurasian community: the Macanese.

Do they have a special cuisine?

‘Macanese cuisine’ is a mix of southern Chinese and Portuguese ingredients and cooking techniques.

And did you know that Macau is the most successful gaming capital on earth?

The city’s booming casino industry accounts for around 80% of its economy.

It remains the only place in China where gambling is legal.

But there is life beyond Macau’s casinos.

Indeed, in 2005, the Historic Center of Macau was inscribed on the UNESCO World Heritage list and includes over 20 locations that exhibit the unique co-existence of two contrasting cultures: Chinese and Portuguese.

Would you like to know more?

Go to the Macao Government Tourism Office and discover it!

### Tonga script

Which countries welcome the new day first?

The International Date Line is an imaginary line on Earth that separates two consecutive calendar days, and it is drawn under the Pacific Ocean, near the 180-degree meridian.

For the interest of some of the Polynesian countries, it goes through, the legal or local hour and the date could pertain to the one in the other hemisphere.

One of those countries is Tonga.

Would you like to know more about it?

Keep watching!

The Kingdom of Tonga is an archipelago made up of 177 islands, of which 45 are inhabited.

Its total surface area is about 750 km^2^, scattered over 700,000 km^2^ in the southern Pacific Ocean.

The biggest and most populated island is Tongatapu.

We could talk for hours about it, but let us keep it short with five interesting facts.

Captain James Cook referred to Tonga as the ‘Friendly Islands’ because the indigenous people gave him and his crew a warm welcome.

What language do they speak?

The two official languages in Tonga are English and Tongan, a West Polynesian language that belongs to the Oceanic branch of the Austronesian language family.

It is closely related to Wallisian, Niuean, and Hawai'ian.

English is used in education and work, while Tongan is used in daily life.

Did you know that the postal seals or stamps of Tonga are highly valued by collectors?

Especially those that are colorful and limited edition.

Tonga issued the first auto-adhesive stamps in the world.

And be careful when you walk around the islands, because the most common cause of premature death in Tonga is the fall of a coconut on the head.

The coconut ripens all year long, and there are coconut trees everywhere.

Last, but not least, Tonga is home to the South Pacific’s equivalent of Stonehenge.

It is called Ha’amonga ‘a Maui or Maui’s Burden.

Would you like to know more about it?

Go to Tourism Tonga and discover it!

## Abbreviations

ANOVA: Analysis of variance
AOI: Areas of Interest
CLT: Cognitive Load Theory
CTML: Cognitive Theory of Multimedia Learning
L1: Native language
L2: Second language
M: Mean
N: Number
SD: Standard Deviation
